# Elevated temperature drives a shift from selfing to outcrossing in the insect-pollinated legume, faba bean (*Vicia faba*)

**DOI:** 10.1093/jxb/erw430

**Published:** 2016-12-07

**Authors:** Jacob Bishop, Hannah E. Jones, Donal M. O’Sullivan, Simon G. Potts

**Affiliations:** 1 School of Agriculture, Policy, and Development, University of Reading,RG6 6AR, UK.

**Keywords:** Allogamy, autogamy, climate change, extreme weather, heat stress, insect pollination, plant–climate interactions, plant–pollinator interactions, *Vicia faba*.

## Abstract

Climate change can threaten the reproductive success of plants, both directly, through physiological damage during increasingly extreme weather events, and indirectly, through disruption of plant–pollinator interactions. To explore how plant–pollinator interactions are modified by extreme weather, we exposed faba bean (*Vicia faba*) plants to elevated temperature for 5 d during flowering, simulating a heatwave. We then moved the plants to flight cages with either bumblebees or no pollinators, or to two field sites, where plants were enclosed in mesh bags or pollinated by wild insect communities. We used a morphological marker to quantify pollen movement between experimental plants. There was a substantial increase in the level of outcrossing by insect pollinators following heat stress. Proportion outcrossed seed increased from 17 % at control temperature, to 33 % following heat stress in the flight cages, and from 31 % to 80 % at one field site, but not at the other (33 % to 32 %). Abiotic stress can dramatically shift the relative contributions of cross- and self-pollination to reproduction in an insect pollinated plant. The resulting increases in gene flow have broad implications for genetic diversity and functioning of ecosystems, and may increase resilience by accelerating the selection of more stress-tolerant genotypes.

## Highlight

We show for the first time that abiotic stress during flowering can dramatically increase the proportion of progeny produced by outcrossing in a mixed-mating, insect-pollinated plant.

## Introduction

Climate change and associated extreme weather events can threaten plant reproductive success directly, through physiological damage at key development stages (Porter *et al.*, 2014), and indirectly, through disruption of plant–pollinator interactions (Settele *et al.*, 2016). An estimated 88% of all flowering plant species (Ollerton *et al.*, 2011) rely to some extent on pollination by an animal vector. Recent changes in climate have already caused distribution shifts in insect pollinator species (Kerr *et al.*, 2015) and this is projected to continue into the future (Rasmont *et al.*, 2015). This has the potential to cause widespread disruptions of plant–pollinator interactions, as, for instance, plants and their pollinators become mismatched in time and space due to their different responses to warming (Hegland *et al.*, 2009; Giannini *et al.*, 2013; Polce *et al.*, 2014). Climate change could therefore threaten genetic diversity and functioning in natural ecosystems (Eckert *et al.*, 2010; Gómez *et al.*, 2011; Vanbergen *et al.*, 2014), and could exacerbate existing pressures facing the insect-pollinated crops that make up around a third of our diet (Klein *et al.*, 2007).

Climate change encompasses not only gradual increases in global average temperature, but also increases in the frequency, severity, and duration of extreme weather events (Hansen *et al.*, 2012; Seneviratne *et al.*, 2012). Very little is known about the effects of these extreme weather events such as heat waves on the animal pollination of plants. This is surprising as heat waves (and resulting heat stress) in particular are expected to limit the future increases in food production (Deryng *et al.*, 2014) that are required to feed a growing global population (Godfray *et al.*, 2010).

Some plant species are known to produce closed, obligate self-pollinating (cleistogamous) flowers when subjected to abiotic stress (Culley and Klooster, 2007). This reproductive strategy may provide some reproductive assurance; closed flowers may have lower internal temperatures than open flowers, providing developing gametophytes with some protection under moderate stress (e.g. in rice; Koike *et al.*, 2015), and seed production is then less dependent on the presence of insect pollinators (e.g. in wild petunia; Munguía-Rosas *et al.*, 2012). In contrast to this strategy, we recently demonstrated that heat stress can make reproduction in faba bean (*Vicia faba* L.), a mixed-mating legume, dramatically more dependent upon insect pollination (Bishop *et al.*, 2016*a*). Fertilization in faba bean occurs through a combination of three mechanisms; in complete absence of insect pollinators (autofertility); requiring an insect to disrupt a physical barrier between the stigma and anthers (tripping; Kambal *et al.*, 1976); or through outcrossing. The mechanistic basis of our observed change in plant–pollinator interaction is unknown, but it is probably due to a deficiency in self pollen being compensated by an increase in outcrossed pollen. Pollen is typically more sensitive to stress than female gametes during development; hand pollination experiments in common bean (Gross and Kigel, 1994), oilseed rape (Young *et al.*, 2004), tomato (Peet *et al.*, 1998), and wheat (Saini and Aspinall, 1982; Briggs *et al.*, 1999) have demonstrated that while flowers may be unable to self-pollinate following stress, they can remain capable of reproduction if provided with fertile pollen (Table 1).

**Table 1.** Summary of experiments that have made crosses of pollen by hand between heat-stressed (HS) and control (C) plants HS outcrossing refers to a HS plant that has received C pollen. Selfing here refers either to a plant allowed to self-pollinate, or to the transfer of pollen between plants of the same temperature treatment. Effect sizes given are the percentage of total flowers setting seed, and are not relative to a control treatment. Studies marked with an asterisk used emasculated or male-sterile lines as the pollen recipient.

Study	Species	Timing of stress	Cross	Seed set (%)
Peet *et al.* (1998)*	Tomato (*Solanum lycopersicum*)	Floral development until maturity	C selfing HS selfing HS outcrossing	100 0 73
Young *et al.* (2004)*	Oilseed rape (*Brassica napus*)	Anthesis	C selfing HS selfing HS outcrossing	79 8 68
Gross and Kigel (1994)	Common bean (*Phaseolus vulgaris*)	Sporogenesis	HS selfing HS outcrossing	0 81
		Anthesis and early pod development	HS selfing HS outcrossing	31 31
Saini and Aspinall (1982)*	Wheat (*Triticum aestivum*)	Sporogenesis	C selfing HS selfing HS outcrossing	89 42 58
Briggs *et al.* (1999)	Wheat	Moisture stress (MS) from early booting throughout anthesis.	C selfing MS selfing MS outcrossing	76 9 48

In this study, we investigated changes in the outcrossing level and reproductive success of faba bean in response to heat stress and insect pollination treatments. We exposed experimental plants to heat stress for a 5 d period during flowering, then immediately exposed the plants to insect pollination at two field sites with wild insect pollinator communities, or with domesticated bumblebees in a tightly controlled flight cage experiment. Temperature treatments were designed to simulate heat stress events that are likely to become a common occurrence during the summer months in Europe by 2050 (Fischer and Schär, 2010), particularly if co-occurring with reduced soil moisture that can lower the temperature threshold at which plants experience heat stress (Lobell *et al.*, 2011). We identified changes in outcrossing level using hilum colour (a scar left on each bean by the stalk that attached it to the pod wall) as a phenotypic marker which is controlled by a single genetic locus in which the black allele is dominant over the white (Gasim *et al.*, 2004). We hypothesized that outcrossing by insect pollinators would increase following heat stress, and correspond to enhanced reproductive resilience to stress.

## Materials and methods

### Cultivars and growing conditions

The experimental cultivar, Wizard, was homozygous recessive, resulting in a white hilum, and the non-stressed pollen donor cultivar, Buzz, was homozygous dominant, resulting in a black hilum. Both cultivars have the same flower morphology and are UK commercial cultivars (Wherry & Sons Ltd). We could identify any experimental Wizard progeny that produced black hilum beans as the result of an insect pollinator transferring pollen from a Buzz plant (e.g. intervarietal outcrossing). It was, however, not possible to identify changes in pollen movement between flowers on the same plant, or between experimental Wizard plants. For this reason, while reliably comparing between treatments our method will have underestimated total levels of outcrossing (Suso *et al.*, 2001) as it was not possible to differentiate between experimental progeny sired by self-pollen, or pollen from other Wizard plants (e.g. selfing versus intravarietal outcrossing). Thus, for the purposes of this paper, selfing refers to within-flower self-pollination (through either autofertility or tripping), pollination between flowers on the same Wizard plant, and between-Wizard pollination, while outcrossing refers to only between-cultivar cross-pollination.

Initial growing conditions for experimental plants were similar for both the field and flight cage experiments. Plants were grown at the Plant Environment Laboratory (now relocated to the Crop and Environment Laboratory), University of Reading, UK (51 27'N lat, 00 56'W long) in plastic pots (180 mm diameter; 4 litre volume) containing vermiculite, gravel, sand, and compost at a ratio of 4:4:2:1, mixed with 2 kg m^−3^ Osmocote slow-release granules (LBS Horticulture). Three seeds were sown per pot on 29 January 2014 for the cage experiment and on 24 March 2014 for the field experiment, and thinned to one plant per pot when three leaf-pairs were unfolded. Plants were randomly assigned to temperature and pollination treatments in both experiments and maintained in a fully enclosed polytunnel until four leaf-pairs had unfolded on the majority of plants, at which point they were randomly distributed in a holding cage. Plants were watered at least daily, either by automatic drip irrigation (cage experiment) or by hand-watering twice daily (during the field pollination treatment) to maintain field capacity throughout experiments.

### Temperature treatments

We exposed the experimental Wizard plants to either heat stress (30/24 °C; day/night temperature, 16 h photoperiod) or control (20/14 °C; same photoperiod) temperature treatments for 5 d during floral development and anthesis using four 1.37 × 1.47 m Saxcil controlled-environment chambers. Treatments began on 16 May 2014 and 22 June 2014 for the cage and field experiment, respectively. We used two replicate cabinets for each temperature treatment in each experiment. We randomized the use of different cabinets among temperature treatments across experiments. Light levels were maintained at 650 µmol m^−2^ s^−1^, carbon dioxide at 385 mg l^−1^, and relative humidity at 85 ± 20% (flight cage experiment) and 90 ± 20% (field experiment). Drip irrigation was applied once (control) or twice (heat stress) daily during temperature treatments to maintain field capacity evenly in both treatments and avoid interactions between heat and drought stress. No temperature treatments were applied to the pollen-donor Buzz plants in either experiment. Following temperature treatments, plants were immediately transferred to the pollination treatments in flight cages or in the field.

### Pollination treatments Cage experiment

There were two main pollination treatments, insect pollination and pollinator exclusion. An additional subset of plants were hand-tripped (see below). We used 10 flight cages in total, located outside at the Plant Environment Laboratory. Each flight cage measured 2.5 × 2.5 × 2 m and was built from 1.33 mm aperture polyethylene mesh suspended inside a metal frame. The insect pollination treatment consisted of five cages containing commercially produced colonies of *Bombus terrestris audax* colonies, positioned centrally. While *B. terrestris* is known to exhibit nectar robbing in faba bean, it was the only commercially available bumblebee, and is an effective pollinator of faba bean in flight cage experiments (Garratt *et al.*, 2014; Bishop *et al.*, 2016*a*). The exclusion treatment consisted of five cages that did not contain bumblebee colonies. In each cage, plants were arranged randomly within concentric circles of 10 Buzz plants, 16 Wizard plants, and 22 Buzz plants to ensure that each Wizard plant was adjacent to the same number of pollen-donor Buzz plants. Pollination treatments began after heat stress on 21 May 2014, bumblebee colonies were later removed at the end of flowering, and plants were retained in flight cages until senescence. Hand-tripping treatments were conducted on four plants in each cage (one plant from each cabinet) in addition to the main treatments. They were designed to measure any differences in reproduction between floral visitation with no pollen transfer (selfing via tripping), and floral visitation with the potential for outcrossed pollen transfer (a bee visit). All open flowers on these plants were manually tripped every 3 d during flowering.

#### Pollination treatments - Field experiment

**Fig. 1.**
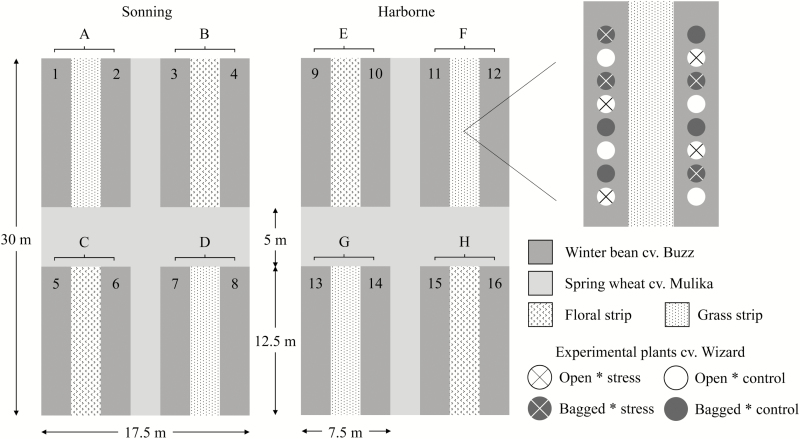
Field plot design. Numbers indicate independent blocks for assessing effect of heat stress and bagging on faba bean yield. Letters indicate independent replicates for management comparisons.

There were two pollination treatments in the field experiment, open pollination, or bagging to exclude insect pollinators. Following the heat stress treatment, on 27 June 2014, experimental plants were distributed in 16 blocks of eight plants (two plants each from four cabinets, of which one plant was bagged and one open; these blocks are indicated with numbers in Fig. 1) within a field of Buzz plants at two sites ~6 km apart (Sonning: 51 48'N, 00 89'E; and Harborne: 51 44'N, 00 94'E). Pollinator exclusion bags were constructed from 1.33 mm aperture mesh that was rolled and stapled to form cylinders ~1 m long that were sealed with cable ties at each end. These bags were moved up the plants periodically to cover all open flowers. Two experiments to investigate the effect of bagging on plant performance did not find a negative impact (Table 2). At the Harborne site there were no flowering faba bean plants in the vicinity (~3 km). At the Sonning site, a 0.05 ha field of the white hilum faba bean cultivar, Fuego, was flowering ~0.2 km away. As maximum foraging ranges of four common bumblebee species have been estimated to be 0.7 km (Knight *et al.*, 2005), sites were assumed to be sufficiently far apart to measure independent pollinator communities. The experimental plants remained at the field sites until 16 July 2014 when they had finished flowering. They were then moved to pollinator exclusion cages at the Plant Environment Laboratory to be protected from pests and automatically irrigated.

**Table 2.** Controls for pollination bagging treatments in the field experiment *F*- and *P*-values are from one-way ANOVAs (yield parameter~bagging treatment). Plant pots in both pollination treatments (bagged and open) had 25 mm holes in their base, through which a cane was driven into the ground. Two 200 mm plant support rings were fitted onto each cane, over which exclusion bags were fitted, or plants were left uncovered for open pollination. For the hand-pollination controls, only results of maximally hand-pollinated nodes were included in mean estimation, three plants were excluded as they had no flowers suitable for hand-pollination.

Control method	Yield parameter	Mean ±SE		*n*	ANOVA test	
		Open	Bagged		*F*-value	*P*-value
Plants either open or bagged within a pollinator exclusion cage measuring 2.5 × 2.5 × 2 m for duration of field pollination treatment.	Yield mass (g) per plant	6.7 ± 1.5	8.9 ± 1.4	32	1.088	0.305
	Bean number per plant	12.2 ± 2.8	13.6 ± 2.6	32	0.129	0.721
	Harvest index per plant	0.64 ± 0.14	0.77 ± 0.11	32	0.582	0.451
Randomized block of eight open and eight bagged control plants at each field site for duration of field pollination treatment. Subset of open flowers hand-pollinated with Buzz pollen on two separate occasions for each flower.	Yield mass (g) per node per plant	1.7 ± 0.3	2.5 ± 0.6	29	1.339	0.257
	Bean number per node per plant	4.2 ± 0.7	4.5 ± 1.0	29	0.038	0.846

### Agronomic treatments and insect pollinator sampling

In the field experiment, we also trialled the use of floral strips as a representative management intervention to enhance insect pollination service to faba bean. Floral strips have been shown to enhance insect pollination in several crops (Garibaldi *et al.*, 2014). We sowed strips of annual flowering plants adjacent to bean-growing areas that synchronzed with the faba bean crop in terms of sowing and flowering time. Four large field plots (indicated as letters in Fig. 1) per site consisted of two strips of Buzz plants (blocks; numbers in Fig. 1) sown at a density of 36 plants m^–2^ each side of a management strip, which was either a grass sward mown prior to pollination treatments (to represent conventional field edge management), or a mixture of flowering annuals that consisted of crimson clover, corn cockle, cornflower, corn marigold, field poppy, and alsike clover at a ratio of 20:9:3:3:3:2 (Cotswold Seeds Ltd). Large field plots were separated by a buffer of spring wheat at least 2.5 m wide.

We assessed insect pollinator density and diversity at each field block using 15 min transect walks (e.g. Dafni *et al.*, 2005), which were performed on 4 d during faba bean flowering. Pollinators in a 2 m area in front of and to the side of the observer were identified as honey-bees, solitary bees, or to a bumble-bee morphological group (*Bombus terrestris/lucorum*; *B. pascuorum*; *B. lapidarius*, *B. hortorum*; *B. hypnorum*; *B. pratorum*). While presented pollinator species counts therefore probably under-represent the actual number of species, they act as a useful comparison between sites and management treatments.

### Data collection

The number and mass of seeds produced by each experimental Wizard plant in 2014 were assessed at senescence. Only seeds from stems present prior to stress were counted, as some plants in the field experiment produced additional stems that grew outside of the exclusion bags. Plants were harvested individually, and seed pods were air-dried at room temperature for 2 weeks until constant mass, before recording seed number and mass per plant. High temperature oven drying was not possible as it was necessary to sow experimental progeny to determine their paternity. The moisture content of eight random samples from different treatments was determined using a moisture analyser (model AP6060; Sinar Technologies) and found to have a mean of 12.4% and range of 0.6%.

Wizard progeny from both experiments were sown in 2015 to determine the number of plants that produced seeds with either black or white hila. Only seeds from stems that were present prior to stress were sown. Progeny of plants in the flight cage pollination treatments (160 parents, *n*=4097) were drilled in 40 plots (corresponding to four cabinets×10 flight cages); with progeny being randomly sampled using a riffle divider if they exceeded 120 per plot. Progeny of plants in the field experiment (128 parents, *n*=2038) were hand-sown, and mapped such that the progeny of each experimental plant could be identified. Each plant was examined individually for hilum colour, rather than at bulk harvest, to avoid potential heterotic effects caused by ‘outcrossed’ progeny producing a greater number of seeds (Suso *et al.*, 1995).

### Statistical analysis

Parameters were analysed using R statistical software (version 3.2.2, R Core Team, 2015). Generalized linear, and linear mixed effects models were used to address the repeated measures at the level of the plants, per cage, cabinet, or field block, using the lme4 package (Supplementary Table S1 at *JXB* online; Bates *et al.*, 2014). Data at the level of the plant were analysed, with the exceptions of (i) the cage outcrossing variable which was analysed using data specific to a cage×cabinet combination (four cabinets×five pollination cages=20 combinations) and (ii) insect pollinator sampling which was analysed using averages for each block, with mainplot as a random effect, because management treatments were at the mainplot scale.

Counts of seeds were analysed using models with a negative binomial error distribution. The response variable in outcrossing analyses was a two-column matrix combining counts of black hilum and white hilum progeny (successes and failures, respectively). Only counts from insect-pollinated plants were included in the final cross-pollination analyses for simplicity; negligible levels of black hilum progeny were recorded from plants grown in exclusion from insect pollinators and were deemed to be accidental from individual bees gaining access to the enclosures, or impurity in the Wizard seed.

Maximal models were simplified by single term deletions tested with likelihood ratio tests (Shmueli, 2010); terms were dropped if *P* >0.05 (Supplementary Table S1). Residuals were checked for normality and heteroscedasticity, and Poisson or binominal models were checked for overdispersion. Effect sizes provided in the text are model estimates unless otherwise stated (and are calculated as an average across all random effect levels).

## Results

### Outcrossing

**Fig. 2.**
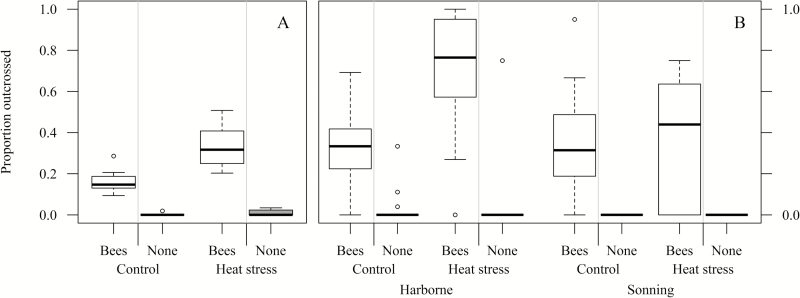
Proportion of outcrossed progeny in flight cage (A) and field (B) experiments. Flight cage data were grouped for each flight cage and cabinet combination (40 data points) while presented field experiment data are at the individual plant level.

The proportion of progeny resulting from outcrossing by insect pollinators increased significantly with heat stress in both the flight cage and field experiments. In the flight cage experiment (Fig. 2A), 16.9% of experimental progeny were outcrossed at control temperature, which increased to 32.8% from heat stress-reated plants (*P* < 0.001).

On average, outcrossing in the field experiment (Fig. 2B) also increased under stress, from 31.8% at control temperature to 55.9% after a 5 d period of heat stress. However, a significant interaction between site and temperature treatment was found (*P*=0.002), with an increase in outcrossing only occurring at the Harborne field site (from 30.7% to 79.9% at Harborne, from 32.9% to 31.9% at Sonning). The outcrossing level did not vary in response to floral strips adjacent to faba bean plots (Table 3).

**Table 3.** Effects of floral strips adjacent to cropping areas on pollinator density and diversity, and on outcrossing level of faba bean

Site	Management type	Pollinator density	Pollinator species count	Proportion outcrossed progeny	
				Control	Stress
Sonning	Floral strip	1.25 ± 0.96	1.13 ± 0.85	0.35 ± 0.18	0.38 ± 0.28
	Grass strip	1.42 ± 0.50	1.21 ± 0.25	0.35 ± 0.27	0.33 ± 0.06
Harborne	Floral strip	1.46 ± 0.63	1.38 ± 0.48	0.34 ± 0.05	0.75 ± 0.29
	Grass strip	1.54 ± 1.13	1.21 ± 0.98	0.32 ± 0.11	0.68 ± 0.13
**Model parameter**					
Site		*P*=0.651	*P*=0.684	–	
Management		*P*=0.733	*P*=0.892	*P=*0.453	
Temp		–	*–*	–	
Site:management		*P*=0.909	*P*=0.682	*P=*0.936	
Temp:management		*–*	*–*	*P=*0.737	

Presented values are the observed mean ± SD, and *P*-values are from likelihood ratio tests (see Supplementary Table S1 for more information).

Presented outcrossing data are from unbagged plants only. Lower level interactions and individual parameters are not included if in a higher level interaction.

### Reproductive success

Overall, insect-pollinated plants produced a greater number and mass of seeds than excluded plants in the flight cage experiment, and at one site in the field experiment. Seed number and seed mass results are presented in Table 4. Open pollinated plants lost fewer seeds after a 5 d period of heat stress than bagged plants at both field experiment sites. Heat stress reduced seed number equally in both pollinated and excluded plants in the flight cage experiment.

**Table 4.** Effects of pollination and temperature treatments on per-plant reproductive success

Experiment or site	Pollination treatments	Seed number		Seed mass (g)	
		Control	Stress	Control	Stress
Sonning	Bees	19.8 ± 12.3	13.1 ± 6.6	9.3 ± 6.4	6.3 ± 3.1
	None	18.6 ± 7.8	9.4 ± 5.3	8.8 ± 5.0	5.2 ± 3.3
Harborne	Bees	26.4 ± 10.8	17.6 ± 8.2	13.2 ± 5.3	8.4 ± 4.2
	None	16.2 ± 6.3	6.2 ± 5.0	9.2 ± 4.1	3.8 ± 3.3
**Model parameter**					
Temp		–		*P*<**0.001**	
Poll:temp		*P*=**0.034**		*P*=0.685	
Poll:site		*P*=**0.003**		*P*=**0.022**	
Temp:site		*P*=0.517		*P*=0.242	
Poll:temp:site		*P*=0.445		*P*=0.959	
Flight cages	Bees	56.0 ± 16.8	34.9 ± 9.9	33.6 ± 7.7	25.5 ± 7.6
	None	29.2 ± 10.3	16.4 ± 5.7	18.4 ± 5.6	10.0 ± 4.8
	Bees+tripping	56.4 ± 24.7	32.1 ± 8.9	31.1 ± 15.1	24.4 ± 5.6
	None+tripping	55.3 ± 20.7	39.4 ± 11.5	29.4 ± 10.2	25.6 ± 7.3
**Model parameter**					
Temp		*P*=**0.005**		*P*=**0.027**	
Poll		*P*<**0.001**		*P*<**0.001**	
Poll:temp		*P*=0.883		*P*=0.847	

Presented values are the observed mean ±SD, and *P*-values are from likelihood ratio tests (see Supplementary Table S1 for more information).

For the flight cage experiment, means and SDs were calculated from data already aggregated across flight cages and cabinets. Abbreviations: poll, pollination treatment, temp, temperature treatment, interactions are indicated by ':'. Lower level interactions and individual parameters are not included if in a higher level interaction.

Bold significance values are significant to *P*<0.05.

Additional hand-tripping treatments in the flight cage experiment produced similar bean numbers to the insect pollinator only treatment, when performed either on plants that were excluded from insect pollinators, or in addition to insect pollination (model with these pollination treatments grouped versus full model, *P*=0.893).

### Pollinator density and diversity

Pollinator density and diversity were slightly higher at the Harborne site, but there were no statistically significant differences between sites or management treatments, and variability in outcrossing level between sites was not explained by management treatments (Table 3).

## Discussion

Recent evidence suggests that gradual changes in climate are likely to modify interactions between insect pollinators and the plants that they visit (Scaven and Rafferty, 2013) due to spatial (e.g. Polce *et al.*, 2014; Kerr *et al.*, 2015) and temporal (e.g. Bartomeus *et al.*, 2011) shifts in pollinators. Climate change is also likely to lead to more extreme weather events (Hansen *et al.*, 2012; Seneviratne *et al.*, 2012). Our study demonstrates that this extreme weather can greatly modify plant–pollinator interactions. We exposed plants to a high temperature stress for 5 d, and observed a dramatic increase in reproduction via outcrossing. A near doubling of outcrossing level following stress occurred both in a tightly controlled flight cage experiment with domesticated insect pollinators, and at a field site with a wild insect pollinator community.

Heat stress during floral development is known to reduce pollen fertility in faba bean (Bishop *et al.*, 2016*b*), such that flowers can become deficient in their own pollen and less able to reproduce by within-flower selfing. A pollen deficiency may also occur indirectly, through reduced anther dehiscence (release of pollen) following stress (Stoddard, 1986). These flowers do not become obligate outcrossers—our hand-tripping treatments showed that remaining self-pollen can be sufficient for a certain level of reproduction if floral visitation is very high (and perhaps unrepresentative of field levels; Garratt *et al.*, 2014). However, given the doubling of outcrossing following heat stress, there is clearly a fertilization advantage of outcrossed pollen when it is present. This could be due to a greater density of outcrossed pollen, faster pollen tube growth, and/or better retention of pods containing outcrossed seeds. Outcrossing of fertile pollen (at least when completed by hand in laboratory studies) has been shown to restore seed set in a range of species (Table 1). We suggest that reproductive recovery by outcrossing is the likely mechanism by which crop yield stability is conferred by insect pollinators (Bishop *et al.*, 2016*a*). It is likely that under field conditions some individual plants retain fertile pollen and act as donors; stress exposure is typically patchy in many environments due to differences in soil depth and soil water-holding capacity.

It has previously been observed that outcrossing occurs more frequently at locations with more dense (Suso *et al.*, 2001) or more diverse (Brittain *et al.*, 2013) pollinator communities, due, for example, to synergistic interactions between pollinating species. Although there were very different responses in terms of outcrossing at our two field sites, we found no quantifiable differences in pollinator community or growing conditions (though different responses could have been due to unobserved pollen quality differences between sites). In addition, the annual floral strips that we trialled in our study did not result in greater pollinator density or diversity, or more outcrossing in the experimental plants. Such management interventions probably require tailoring to specific crop systems, and can take several years to become effective (Blaauw and Isaacs, 2014). This warrants larger scale field experimentation that compares responses at many sites, ideally across a pollinator density and/or diversity gradient, to be better able to predict sites and management strategies that deliver high levels of outcrossing and floral visitation.

**Fig. 3.**
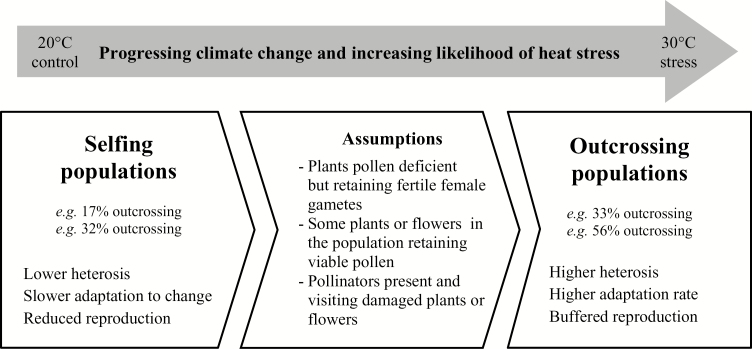
A conceptual diagram for mixed-mating plant species: with minimal climate change there was low likelihood of extreme weather events. With future projected climate change, and even current climate change, this likelihood has increased, leading to greater probability of outcrossing in populations.

Dramatically increased outcrossing following heat stress has broad implications for plant–pollinator interactions in natural ecosystems (summarized in Fig. 3). Extreme weather events that can cause heat stress are already a common occurrence (Hansen *et al.*, 2012), and could already be driving changes in the gene flow of angiosperms, 88% of which require pollination by animals (Ollerton *et al.*, 2011). Greater incidence of heat stress and subsequent enhanced outcrossing could drive rapid ecologically adaptive evolution of mixed-mating species in natural ecosystems, similar to that seen in obligate outcrossing grasses under simulated climate change (Ravenscroft *et al.*, 2015). For this process to occur widely in insect-pollinated plants, it would also require stable pollinator populations and pollination services under future climate change, though there are many uncertainties (Settele *et al.*, 2016).

It is vital that we avoid ‘lose–lose’ scenarios in which plant stress increases, and we lose the resilience that can be provided by insect pollination. For example, in managed systems, agricultural producers could respond to increasingly extreme weather and unstable crop yields by further intensifying production (Nelson *et al.*, 2014). Without thoughtful planning, this could be detrimental to insect pollinator populations and any resilience benefits (e.g. Bishop *et al.*, 2016*a*) that they provide (both in crop fields and in surrounding habitats), due to degradation of floral and nesting resources (Dormann *et al.*, 2008). Instead, we could promote highly species diverse pollinator communities that are functionally resilient under climate change (Bartomeus *et al.*, 2013; Rader *et al.*, 2013). Approaches to achieve this include increasing the provision of feed and nesting habitats in landscapes (Goulson *et al.*, 2015), and the connectivity between habitats within landscapes. Greater incorporation of mass-flowering legume crops in agricultural rotations may form part of this strategy (e.g. Rundlof *et al.*, 2014; but see Holzschuh *et al.*, 2016), and has many additional benefits for sustainable agriculture (e.g. Köpke and Nemecek, 2010).

We have measured a substantial change in the level of outcrossing in faba bean following heat stress. Further experimentation should test (i) applications of this process to cropping systems that utilize managed pollinators; and (ii) the generality of this process (e.g. analyses of outcrossing level of mixed-mating species in long-term stress experiments). There is growing evidence that climate change will reduce plant reproductive success and exacerbate the many existing pressures on pollination services (González-Varo *et al.*, 2013). This demands practises that conserve and best utilize available pollination services (Garibaldi *et al.*, 2014), to ensure that insect pollinator communities are maintained, and their benefits maximized across both agricultural and natural ecosystems.

## Supplementary data

Supplementary data are available at *JXB* online

Table S1. Model simplification tables for establishment of treatment effect sizes and significance.

## Data deposition

Measures of cross-pollination and yield production of faba bean (*Vicia faba* L.) in response to heat stress and insect pollination treatments. University of Reading Research Data Archive. http://dx.doi.org/10.17864/1947.83.

## Authors’ contributions

HEJ, SGP, and JB conceived the work. JB designed and conducted the experiments, analysed and interpreted the data, and wrote the paper, all with advice and comments from HEJ, DOS, and SGP

## Supplementary Material

Supplementary_Table_S1
