# Supplementary material for: Elevated temperature drives a shift from selfing to outcrossing in the insect-pollinated legume, faba bean (Vicia faba)
Source: J Exp Bot. 2016 Dec 7;68(8):2055–63. doi: 10.1093/jxb/erw430 (PMC5429019; doi:10.1093/jxb/erw430)
Supplement: Supplementary_Table_S1 [file erw430_suppl_Supplementary_Table_S1.pdf]

Supplementary data

Bishop *et al.* Elevated temperature drives a shift from selfing to outcrossing in the insect pollinated legume, faba bean (*Vicia faba*).

Table S1 Model simplification tables for establishment of treatment effect sizes and significance. Model parameters are deleted from the maximal model in steps, if a likelihood ratio test comparing the new model with the previous model finds no significant difference in explanatory power (p>0.05), the parameter is left out of the model. Mixed models are distinguished by random effects (in brackets) in the maximal model, candidate models are compared with a $\chi 2$ test. Interactions between terms are indicated using the ":" symbol

A) Outcrossing per cabinet*cage combination, flight cage experiment

**Maximal model:**	Outcrossing (cbind(dark,light)) ~ temperature			
**Error family & link function:**	Quasibinomial, logit			
**Deletion step**	**Parameter**	deviance	**df**	***p*-value**
**1**	Temperature	-38.748	1	<0.001
**Fixed effect in minimal adequate model**		**Estimate**	**SE**	***t*-value**
Control temperature plants with insect pollination		-1.5930	0.1639	-9.718
Effect of heat stress		0.8751	0.2120	4.127

B) Seed number per plant, flight cage experiment

**Maximal model:**	Bean number ~ temperature + pollination + temperature : pollination + (1|cabinet) + (1|cage)			
**Error family & link function:**	Negative binomial, log			
**Deletion step**	**Parameter**	χ^2	**df**	***p*-value**
**1**	Temperature : pollination	0.6595	3	0.8827
**2**	Temperature	7.9157	1	0.004901
	Pollination	38.524	3	<0.001
**3**	Pollination simplified to either exclusion or any one of three pollination treatments	0.2254	2	0.8934
**Fixed effect in minimal adequate model**		**Estimate**	**SE**	***z*-value**
Control temperature plants excluded from pollination and no tripping		3.33848	0.09536	35.01
Heat stress		-0.50327	0.10121	-4.97
Insect pollination, insect pollination + hand tripping, exclusion + hand tripping		0.70716	0.10288	6.87

C) Yield mass per plant, flight cage experiment

**Maximal model:**	Yield mass ~ temperature + pollination + temperature : pollination + (1|cabinet) + (1|cage)			
**Error family & link function:**	Gaussian, identity			
**Deletion step**	**Parameter**	**χ^2**	**df**	***p*-value**
**1**	Temperature : pollination	0.8092	3	0.8473
**2**	Temperature	4.8991	1	0.02687
	Pollination	40.925	3	<0.001
**3**	Pollination simplified to either exclusion or any one of three pollination treatments	0.7657	2	0.6819
**Fixed effect in minimal adequate model**		**Estimate**	**SE**	***t*-value**
Control temperature plants with insect pollination, without hand tripping, with hand tripping, or excluded with hand tripping		32.533	2.502	13.002
Heat stress		-7.468	3.420	-2.184
Exclusion from insect pollinators and no tripping		-14.580	1.733	-8.412

D) Outcrossing per plant, field experiment

**Maximal model:**	Outcrossing (cbind(dark,light)) ~ temperature + site + temperature : site + (1|block) + (1|cabinet) + (1|observation level random effect)			
**Error family & link function:**	Binomial, logistic			
**Deletion step**	**Parameter**	**χ^2**	**df**	***p*-value**
**1**	Temperature : site	9.919	1	0.002
**Fixed effect in minimal adequate model**		**Estimate**	**SE**	***z*-value**
Control temperature plants at Harborne site		-0.813	0.340	-2.388
Heat stress		2.195	0.479	4.575
Sonning site		0.101	0.489	0.207
Heat stress : Sonning site		-2.239	0.689	-3.249

E) Seed number per plant, field experiment

**Maximal model:**	Bean number ~ temperature + pollination + site + temperature : pollination + temperature : site + pollination : site + temperature : pollination : site + (1|block) + (1|cabinet)			
**Error family & link function:**	Negative binomial			
**Deletion step**	**Parameter**	**χ^2**	**df**	***p*-value**
**1**	Temperature : pollination : site	0.583	1	0.445
**2**	Temperature : pollination	4.472	1	0.034
	Temperature : site	0.421	1	0.517
	Pollination : site	8.562	1	0.003
**Fixed effect in minimal adequate model**		**Estimate**	**SE**	***z*-value**
Control temperature plants that were bagged at Harborne site		2.7213	0.1177	23.129
Heat stress		-0.8150	0.1412	-5.771
Insect pollination		0.5544	0.1614	3.435
Sonning site		0.2638	0.1425	1.851
Heat stress : insect pollination		0.4044	0.1934	2.091
Insect pollination : Sonning site		-0.5593	0.1927	-2.903

F) Yield mass per plant, field experiment

**Maximal model:**	Yield mass ~ temperature + pollination + site + temperature : pollination + temperature : site + pollination : site + temperature : pollination : site + (1|block) + (1|cabinet)			
**Error family & link function:**	Gaussian, identity			
**Deletion step**	**Parameter**	χ^2	**df**	***p*-value**
**1**	Temperature : pollination : site	0.0026	1	0.9595
**2**	Temperature : pollination	0.165	1	0.6846
	Temperature : site	1.3716	1	0.2415
	Pollination : site	5.3027	1	0.02129
**3**	Temperature	11.902	1	0.00056
	Pollination : site	5.2322	1	0.02217
**Fixed effect in minimal adequate model**		**Estimate**	**SE**	***t*-value**
Control temperature plants that were bagged at Harborne site		8.5891	0.9057	9.484
Heat stress		-4.1896	0.7740	-5.413
Insect pollination		4.3003	1.0945	3.929
Sonning site		0.5250	1.1580	0.453
Insect pollination : Sonning site		-3.5342	1.5479	-2.283

G) Pollinator density, field experiment. NB. Mainplots are indicated with letters in figure 1, main text. Management treatments were conducted at this mainplot level.

**Maximal model:**	Pollinator number ~ management + site + management : site + (1|mainplot)			
**Error family & link function:**	Gaussian, identity			
**Deletion step**	**Parameter**	χ^2	**df**	***p*-value**
**1**	management : site	0.013	1	0.909
**2**	management	0.117	1	0.733
	site	0.207	1	0.649
**3**	site	0.205	1	0.651
**Fixed effect in minimal adequate model**		**Estimate**	**SE**	***t*-value**
Intercept		1.42	0.19	7.431

H) Pollinator species count. NB. Mainplots are indicated with letters in figure 1, main text. Management treatments were conducted at this mainplot level.

**Maximal model:**	Pollinator species number ~ management + site + management : site + (1|mainplot)			
**Error family & link function:**	Gaussian, identity			
**Deletion step**	**Parameter**	χ^2	**df**	***p*-value**
**1**	management : site	0.168	1	0.682
**2**	management	0.019	1	0.892
	site	0.166	1	0.683
**3**	site	0.166	1	0.684
**Fixed effect in minimal adequate model**		**Estimate**	**SE**	***t*-value**
Intercept		1.23	0.16	7.743

I) Outcrossing per plant, insect pollinator sampling scale. NB. Mainplots are indicated with letters and blocks with numbers in figure 1, main text. Management treatments were conducted at this mainplot level.

**Maximal model:**	Outcrossing (cbind(dark,light)) ~ management + site + temp + management : site + management : temp + site : temp + management : site : temp + (1|cabinet) + (1|mainplot/block) + (1|observation level random effect)			
**Error family & link function:**	Binomial, logistic			
**Deletion step**	**Parameter**	χ^2	**df**	***p*-value**
**1**	management : site : temp	0.002	1	0.966
**2**	management : site	0.006	1	0.936
	site : temp	9.863	1	0.002
	management : temp	0.111	1	0.739
**3**	management : temp	1.113	1	0.737
	site : temp	9.859	1	0.002
**4**	management	0.563	1	0.453
	site : temp	9.889	1	0.002
**Fixed effect in minimal adequate model**		**Estimate**	**SE**	***z*-value**
Control temperature plants that were bagged at Harborne site		-0.813	0.340	-2.389
Heat stress		2.195	0.489	4.574
Sonning site		0.102	0.489	0.207
Heat stress : Sonning site		-2.238	0.689	-3.249
